# Human Midbrain Organoids Enriched With Dopaminergic Neurons for Long‐Term Functional Evaluation

**DOI:** 10.1111/cpr.70005

**Published:** 2025-02-20

**Authors:** Xinyue Wang, Gaoying Sun, Mingming Tang, Da Li, Jianhuan Qi, Chuanyue Wang, Yukai Wang, Baoyang Hu

**Affiliations:** ^1^ Key Laboratory of Organ Regeneration and Reconstruction, State Key Laboratory of Stem Cell and Reproductive Biology, Institute of Zoology Chinese Academy of Sciences Beijing China; ^2^ University of Chinese Academy of Sciences Beijing China; ^3^ Department of Otolaryngology‐Head and Neck Surgery, Shandong Provincial ENT Hospital Shandong University Jinan China; ^4^ Institute for Stem Cell and Regeneration Chinese Academy of Sciences Beijing China; ^5^ Beijing Institute for Stem Cell and Regenerative Medicine Beijing China

**Keywords:** dopaminergic neurons, electrophysiology, human embryonic stem cells, midbrain organoids, neural network

## Abstract

Human midbrain organoids with functional dopaminergic (DA) neurons are invaluable for the therapeutic development of Parkinson's disease (PD). However, current methods face significant limitations, including challenges in generating pint‐sized organoids enriched with DA neurons and the lack of robust functional assays for efficiently evaluating neural networks over extended periods. Here we present an innovative approach that combines developmental patterning with mechanical cutting to produce small midbrain organoids, with diameters less than 300 μm, suitable for long‐term evaluation, along with a comprehensive functional assay system consisting of calcium transient assay, neurite extension assay, and multielectrode array (MEA) assay. Radial cutting of organoids into four to eight portions according to their sizes at the appropriate developmental stage significantly increases the yield of viable organoids while reducing necrotic cell regions. Using the functional assay system, we demonstrate that DA neurons within the organoids extend long projections, respond to dopamine stimulation, and form neural networks characterised by giant depolarising potential‐like events. Our approach supports the generation of midbrain organoids and PD models that can be used for long‐term functional testing.

## Graphical abstract

We optimised a protocol that combines developmental patterning with mechanical cut to produce pint‐sized midbrain organoids, with diameters less than 300 μm, suitable for long‐term evaluation, along with a comprehensive functional assay system consisting of calcium transient assay, neurite extension assay, and multielectrode array assay.

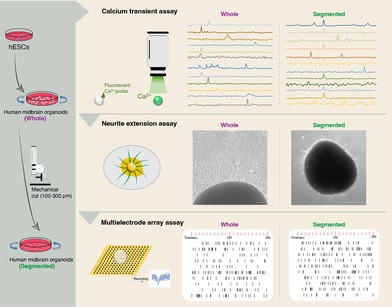

## 1 Introduction

Dopaminergic (DA) neurons are primarily located in the ventral midbrain, which is involved in the regulation of complex behaviours and locomotion [1, 2, 3, 4, 5]. Malfunctioning dopamine neural circuits, such as nigrostriatal and mesolimbic pathways, are central to neurological diseases like Parkinson's disease (PD) [6, 7, 8, 9]. Our understanding of the unique development and functional regulation of human DA neurons, their selective vulnerability to pathogenic factors, and their response to environmental changes during disease progression remains limited [10]. It has hindered the effective development of therapeutic interventions. A novel human model that effectively mimics the physiological and pathological conditions of DA neurons and PD is urgently needed.

Human organoid technology, derived from human pluripotent stem cells (hPSCs), holds great promise for studying human brain development and neurological diseases [11, 12, 13, 14, 15]. Three‐dimensional (3D) self‐organising organoids, including midbrain organoids containing DA neurons, not only replicate cellular composition but also exhibit functionality in vitro [11, 16, 17, 18, 19]. Traditional approaches for generating hPSCs‐derived organoids involve modulating key signalling pathways to recapitulate developmental trajectories, which often require extended culture periods [20]. During extended culture, midbrain organoids often become heterogeneous in size, with some developing into oversized spheres of irregular morphology. As they grow beyond a certain size, these organoids typically cease growing and exhibit core necrosis, likely due to limited oxygen or nutrient availability [21, 22]. Therefore, long‐term culture introduces significant drawbacks that limit the utility of these technologies for practical screening and evaluation [23]. Attempts to dissociate the oversized organoids and reassemble the single cells into spheres usually compromise the survival of the DA neurons, which significantly alters the nature of the midbrain organoids. These limitations affect both the yield and quality of midbrain organoids and often lead to batch‐to‐batch variability, further complicating their applications [24]. In addition, to demonstrate the in vivo function of midbrain, essential functional assays, especially those that can detect the synergistic neural network in midbrain organoids, are urgently needed.

In this study, we optimised a protocol for generating midbrain organoids enriched with DA neurons from human embryonic stem cells (hESCs). Building on the self‐assembled spheroids that sequentially mimic midbrain development and DA neuron differentiation, we introduced a mechanical cut step to divide large organoids into pint‐sized pieces with diameters less than 300 μm. These pieces reorganised into a secondary generation of organoids, which resumed growth and developed spherical shapes. Using the functional assay sets composed of calcium transient assay, neurite extension assay, and multielectrode array (MEA) assay, we demonstrated that the resulting secondary organoids exhibit appropriate neural activity and a functional neural network. This optimised protocol offers significant advantages for studying DA neuron‐related disease mechanisms and for drug screening applications.

## 2 Materials and Methods

### 2.1 hESCs Lines and Culture

Human embryonic stem cell H9 cell line was used in this study. H9 ESCs in this article are from WiCell. The study was approved by the Ethical Committee, Institute of Zoology, Chinese Academy of Sciences, and all procedures in this study were completed under the guidelines of the Institute of Zoology, Chinese Academy of Sciences. H9 hESCs were cultured on 1% Matrigel (Corning)‐precoated 6‐well plates and routinely maintained in Essential 8 medium (Invitrogen) in a 37°C and 5%CO_2_ incubator. Cells were passaged at 80% confluence using 0.5 mM EDTA in the form of clones. Cells were tested for mycoplasma every time before use.

### 2.2 Generation of Human Midbrain Organoids

Human midbrain organoids were differentiated following the developmental trajectory of DA neurons. When hESCs reached 80% confluency, we used Accutase (Invitrogen) to dissociate clones into single cells and seeded them into a V‐bottom low adhesive 96‐well plate. A density of 5000 cells per well in E8 medium containing 10 μM Y27632 (Millipore) was used, and we defined it as day 0 (D0). After 4 days of incubation, the medium was exchanged with neuronal induction medium (NIM). NIM was N2B27 medium supplemented with 100 nM LDN193189 (Selleck), 10 μM SB431542 (Selleck), 0.6 μM CHIR99021 (Stemgent), 300 ng/mL sonic hedgehog (SHH C25II, Peprotech), and 10 μM Y27632. N2B27 medium contained 50% DMEM/F12 (Gibco) and 50% Neurobasal medium supplemented with 1X N2 (Gibco), 1X B27 (Gibco), 1X NEAA (Gibco), 1X Glutamax (Gibco), 0.1 mM β‐mercaptoethanol (Sigma) and 5 μg/mL Insulin (Sigma). On D8, organoids were gently removed to a low adhesive 10‐cm dish and supplemented with NIM to 17 mL, while they were cultured on an orbital shaker from then on. On D15, the medium was changed to progenitor expansion medium (PEM), which is N2B27 medium with 20 ng/mL BDNF (Peprotech), 20 ng/mL GDNF (Peprotech) and 0.2 mM ascorbic acid (AA, Sigma). The fresh medium was fully changed every other day. On D27, the medium was changed to terminal differentiation medium (TDM), namely 20 ng/mL BDNF, 20 ng/mL GDNF, 0.2 mM AA, 0.5 mM dibutyryl cyclic‐AMP (dcAMP, Sigma) and 10 μM DAPT (Sigma) added to N2B27 medium. The medium was changed every 7 days.

### 2.3 Expansion of Segmented Human Midbrain Organoids

Prior to segmentation, human midbrain organoids were incubated in TDM with 10 μM Y‐27632 for 24 h to ensure cell survival. We chose several uniformly sized organoids (on the 35–60 day of differentiation) and put them into a 10‐cm dish with DPBS (Corning). A sterile scalpel blade was used to mechanically cut them into clusters of about 300 μm diameters. Organoids doubled their numbers after each split to ensure every cut was even. Next, we put them back into TDM (with 2 μM Y27632) and cultured them in spinning low adhesive dishes. After 7 days of recovery, we removed the spheres with uneven shapes under the microscope, and the rest continued to recover until at least 14 days before use.

For cellularisation of human midbrain organoids, human midbrain organoids were dissociated in Accutase at 37°C for 15 min. Tubes were flipped about 4 times during dissociation and centrifuged at 2000 rpm for 3 min. Cells were resuspended in the V‐bottom low adhesive 96‐well plate at a density of 5000 cells per well. TDM medium containing 10 μM Y27632 was used.

### 2.4 Immunofluorescence

For whole‐mount immunofluorescence, organoids were fixed in 4% paraformaldehyde (PFA) for 48 h at 4°C, followed by immersion in PBS for 24 h. Next, samples were blocked in block solution for 24 h at 4°C. The block solution was changed to 1% Triton X‐100 (Sigma) and 5% bovine serum albumin (BSA, Sigma) in PBS. Subsequently, samples were incubated in block solution‐diluted primary antibodies at 4°C for 3–5 days according to their size (See Table S1 for details). Then, samples were washed at least three times with 0.1% Triton X‐100 in PBS at 4°C for 24 h in total, followed by incubation in block solution‐diluted secondary antibodies at 4°C for 12 h. Finally, samples were washed with PBS for at least 6 h, and images were captured via ZEISS LSM 880 confocal microscope.

For monolayer‐cultured cell immunofluorescence, samples were fixed in 4% PFA for 30 min at room temperature followed by immersion in PBS for 1 h. Samples were blocked in 0.5% Triton X‐100 and 5% BSA in PBS for 90 min at room temperature. Subsequently, samples were incubated in block solution‐diluted primary antibodies at 4°C for 12 h (See Table S1 for details). Then samples were washed at least three times with 0.1% Triton X‐100 in PBS at room temperature for 1 h in all, followed by incubation in block solution‐diluted secondary antibodies at room temperature for 90 min. Finally, samples were washed with PBS for at least 1 h and images were captured via ZEISS LSM 880 confocal microscope.

### 2.5 Bulk RNA Sequencing

The experiment and analysis were performed by Shandong Xiuyue Biol (Jinan, China). We collected organoids on selected differentiation days and extracted RNA using the TRIzol (Invitrogen) method. Every sample contained 1–7 organoids depending on their sizes. For the universality of the results, we collected three samples for each time point and from at least two batches. RNA quality was determined by the NanoPhotometer and RNA Nano 6000 Kit (Agilent Technologies). The cDNA library was constructed using the VAHTS Universal V6 RNA‐seq Library Prep kit and sequenced by Novaseq 6000 S4. RNA expression of genes was represented by fragments per kilobase per million values (FPKM values).

### 2.6 Calcium Transient Assay

Organoids were loaded with 2 μM Fluo‐4 AM (Invitrogen) in TDM and incubated at 37°C for 0.5 h. After washing with PBS, organoids were placed on a laser confocal petri dish and the calcium responses were sampled by ZEISS LSM 880 confocal microscope under 488 nm at sample frequencies of 0.1 Hz and 0.2 Hz. DA neurons were activated by 20 μM dopamine‐HCl (Selleck) for 20 min at room temperature. Fresh TDM was used to wash out the agonistic action. Calcium activity was quantified by relative fluorescence intensity (Δ*F*/*F*0).

### 2.7 Neurites Extension Assay

Matrigel (Corning) was used in the neurite extension experiment. Organoids were transferred to 20–50 μL Matrigel according to their sizes and droplets on cell slides, whereafter a moderate amount of TDM was gently added. Continued culturing for 2 more days and sampling for follow‐up experiments.

### 2.8 Multielectrode Array Assay

MEA is an electrophysiological test system for the detection of extracellular spontaneous field potential of organoids using an electrode model of 256 MEA. Organoids were cultured on Matrigel‐precoated MEA wells for 2 days before testing. Organoids were maintained at 37°C using temperature control devices during the experiment, and data were recorded using Multi Channel Experimenter version 2.15.0 software. DA neurons were activated by 20 μM dopamine‐HCl for 2 min at 37°C. Fresh TDM was used to wash out the agonistic action. Analysis was performed using Multi Channel Analyser version 2.15.0 software.

### 2.9 Cell Counting and Statistical Analysis

For quantification of cells in organoids, samples were dissociated in Accutase at 37°C for 15 min and centrifuged at 2000 rpm for 3 min. Cells were re‐suspended on a 1% Matrigel pre‐coated confocal petri dish in a 37°C and 5%CO_2_ incubator. The monolayer‐cultured cells were fixed after 24 h for the following immunofluorescent staining. ImageJ software was used for cell counting.

Data in this study were statistically analysed using Prism 10 software. Two‐tailed unpaired t tests were used between two groups, and One‐way ANOVA followed by Tukey's multiple comparison test was used between three or more groups. Data were presented as mean ± SEM. *p* < 0.05 was considered statistically significant.

## 3 Results

### 3.1 Midbrain Organoids Follow Developmental Trajectory and Exhibit Long‐Term Survival

**FIGURE 1:**
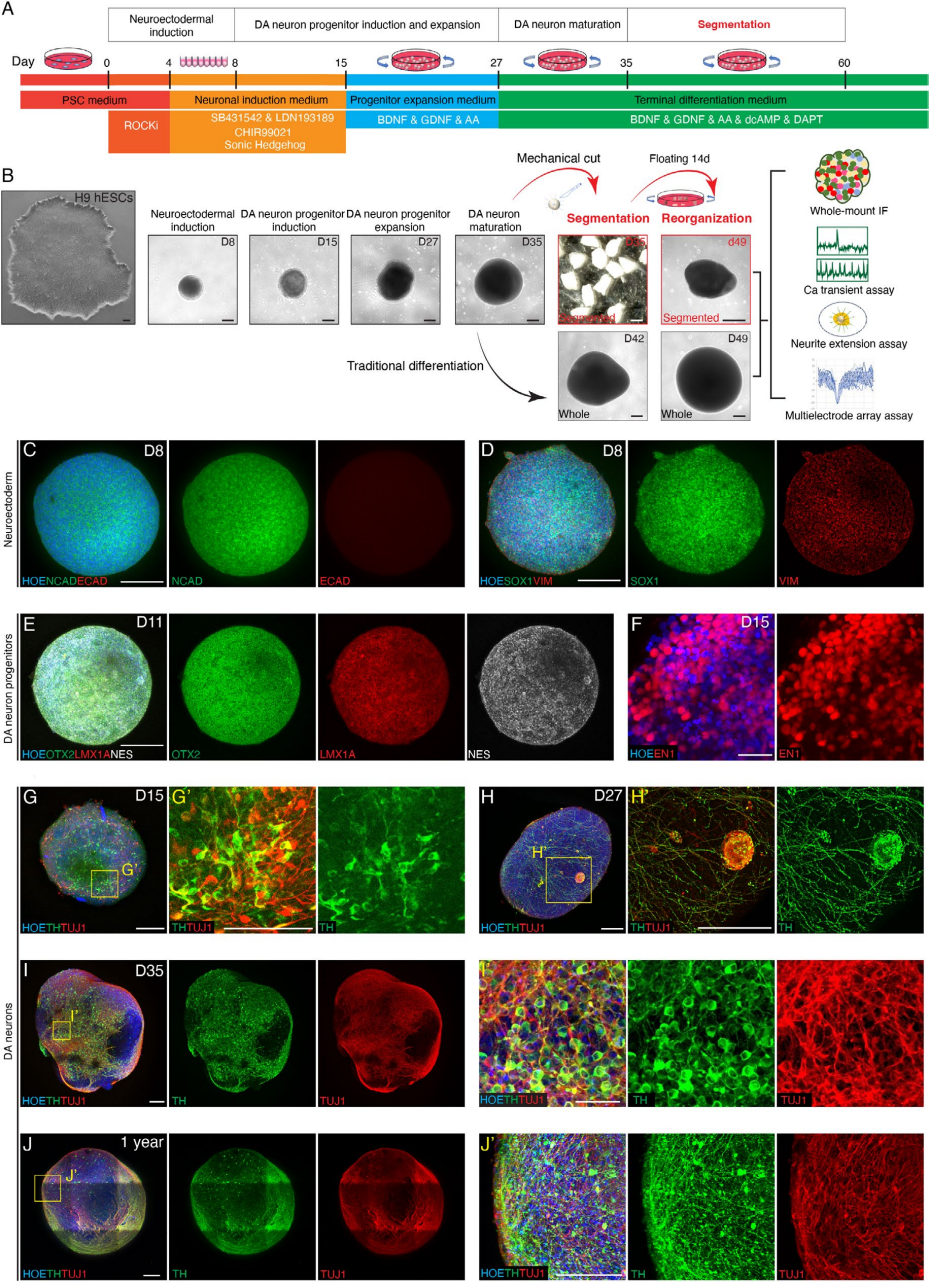
Stepwise patterning of human midbrain organoids using hESCs. (A) Schematic diagram of DA neurogenesis and the segmentation of midbrain organoids for further application. (B) Expanding midbrain organoids through mechanical cut and floating reorganisation. D, differentiation days in vitro of midbrain organoids. d, differentiation days in vitro of segmented midbrain organoids, such as d49 means mechanical cut of midbrain organoids on D35 and floating reorganisation for 14 days. Scale bar, 200 μm. (C and D) Expression of neuroectodermal markers (NCAD, SOX1), endoderm marker (ECAD), mesenchymal cell marker (VIM) on D8. Scale bars, 200 μm (C, D). (E) Representative immunostaining images of neuronal progenitor marker (NES), ventral midbrain marker (OTX2) and floor plate domain marker (LMX1A) on D11. Scale bar, 200 μm. (F) DA neuron progenitor marker (EN1) was positive on D15. Scale bar, 50 μm. (G–I) Wholemount immunostaining of midbrain organoids on D15 (G), D27 (H) and D35 (I) to identify the development of DA neurons (TH‐positive and TUJ1‐positive). Scale bars, 200 μm (G, G', H, H', and I), 50 μm (I′). (J) DA neurons (TH‐positive and TUJ1‐positive) were detected in 1‐year midbrain organoids by wholemount immunofluorescence staining. Scale bars, 200 μm (J, J').

Protocols for midbrain organoid generation typically utilised synchronised activation of the ventralising SHH pathway and the caudalising WNT signalling pathway in hESCs‐derived neuronal ectoderm aggregates (Figure 1A,B). Single cellularised hESCs formed aggregates that grew rapidly. Initially, these organoids expressed high levels of neuroectodermal markers NCAD and SOX1, but not the endodermal marker ECAD (Figure 1C,D). By around day 11, the organoids expressed neuronal progenitor marker NES, ventral midbrain marker OTX2, and floor plate domain marker LMX1A (Figure 1E). From day 15, DA neuron or progenitor markers such as EN1 (61.9% ± 2.14%) began to appear (Figures 1F and S1A,B). At this stage, only a few tyrosine hydroxylase (TH)‐ and TUJ1‐positive cells with short and interrupted neurites were present, indicating immaturity (Figure 1G).

By D27, neuronal integrity improved, with low‐density neurites surrounding the organoid surface (Figure 1H), and small clusters of neurons scattered within the organoids (Figure 1H'). After 5 weeks of expansion, organoid growth slowed significantly, accompanied by a sharp decline in cell cycle markers, signifying neuronal differentiation (Figure S1C). Cells dissociated from D35 organoids were cultured in a monolayer pattern for cell counting. There were about 70.94% ± 0.14% TH‐positive DA neurons on D35 (Figure S1D). At this stage, TH‐ and TUJ1‐positive neurites densely wrapped the surface of the organoids, forming clear, connected neuron body boundaries and networks, indicative of mature DA neurons (Figure 1I). These midbrain organoids can survive in vitro for over 1 year with intact morphology and considerable DA neurons (Figure 1J).

### 3.2 Expansion of Human Midbrain Organoids Through Mechanical Splitting

**FIGURE 2:**
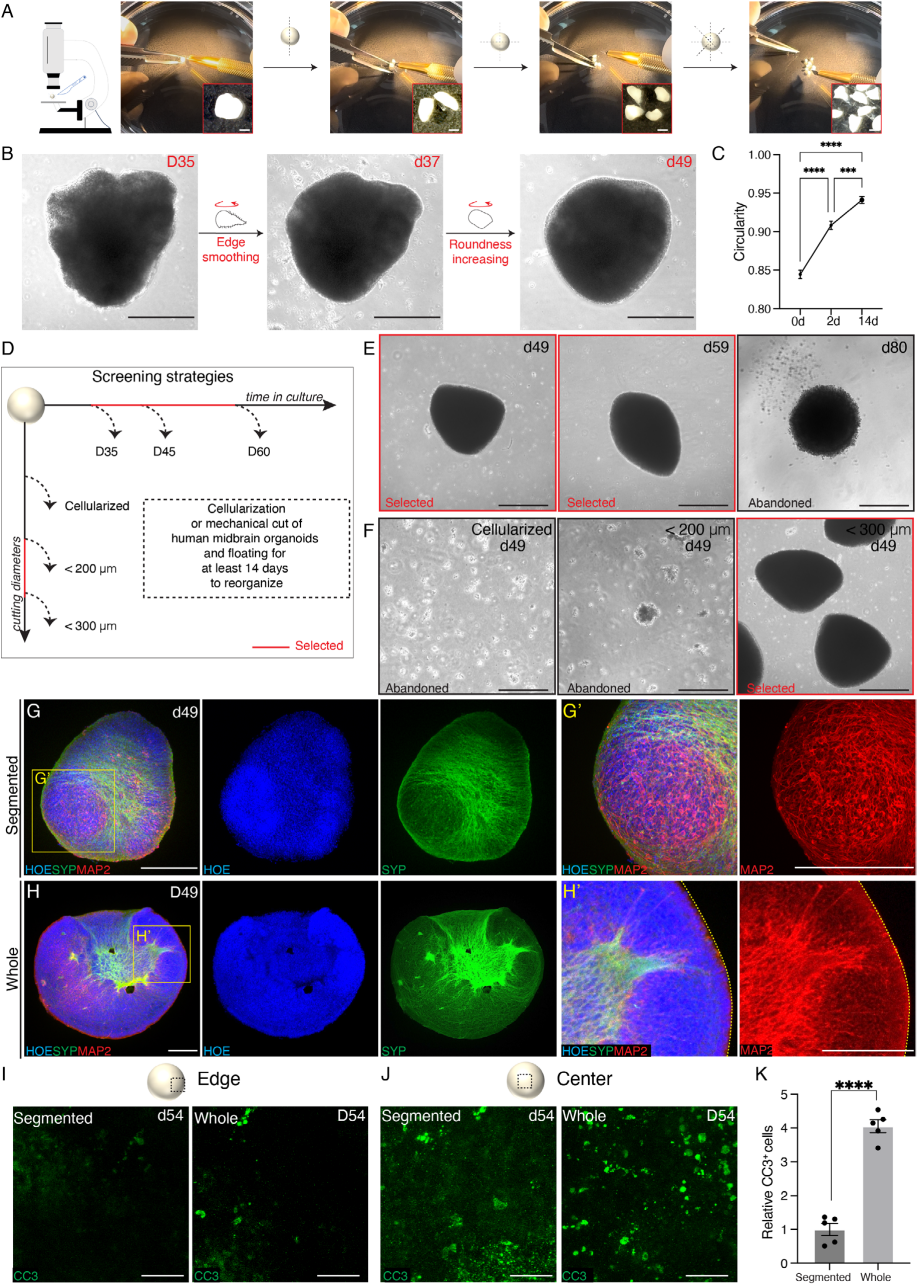
Mechanical cut produced segmented human midbrain organoids with reduced cell apoptosis across whole spheroids. (A) Main steps for the establishment of segmented midbrain organoids derived from whole organoids. Scale bar, 200 μm. (B) Representative brightfield images of the repair process after the mechanical cut of whole organoids on D35. Scale bar, 200 μm. (C) Quantification of the circularity of segmented organoids during the process of recovery. *n* = 4–5 organoids per group, two independent experiments. Data were shown as mean ± SEM. One‐way ANOVA followed by Tukey's multiple comparison test. ****p < 0.0001, ***p = 0.0003. (D) Strategies to choose the most suitable time and sizes for segmentation, with the main changes suggested. (E and F) Representative brightfield images of morphological changes in different handling dates and sizes. Scale bar, 200 μm. (G) Wholemount immunofluorescence staining of maturation neurons (SYP‐positive and MAP2‐positive) in segmented organoids on d49. Scale bar, 200 μm. (H) Immunostaining of midbrain organoids on D49 to identify the existence of functional mature neurons (MAP2‐positive and SYP‐positive). Scale bar, 200 μm (H, H′). (I and J) Representative immunofluorescence images for apoptotic marker (cleaved caspase 3, CC3) in differentiation time matched whole and segmented organoids at different positions (edge or center). Scale bar, 50 μm (I, J). (K) Comparison of apoptotic cells (CC3‐positive) in whole and segmented midbrain organoids. *n* = 4–5 organoids per group, two independent experiments. Data were shown as mean ± SEM. Two‐tailed unpaired *t*‐test. ****p < 0.0001.

Since chemical or enzymatic dissociation can compromise cellular composition, we adopted a mechanical splitting approach to divide large midbrain organoids into small pieces under a stereomicroscope (Figure 2A). Large organoids were precisely cut along the radius to ensure equal division and rapid reorganisation. Fragments were immediately transferred into a serum‐free medium containing a ROCK inhibitor and placed on an orbital shaker to facilitate wound recovery. This process reduced apoptotic cells typically located in the organoid core. After 2 weeks, sharp edges and burrs were repaired, the circularity of segmented organoids was increased, and defined borders were formed (Figure 2B,C).

By analysing their expansion kinetics, we determined the optimal cutting time (days 35–60) and fragment sizes (200–300 μm in diameter) (Figure 2D–F). There were two considerations for the choice of time points. We hope that the time point of mechanical cut cannot affect the normal development of organoids while having the ability to rapidly repair. Both bulk RNA sequencing results and immunostaining results showed that KI67 was significantly decreased at D35, indicating there were almost no proliferating cells in organoids (Figures S1C and S2A). In the meantime, the organoids began to show mature DA neuron morphology on D35 (Figure 1I). Therefore, D35 was chosen as a start point for the cut. We found that cutting the organoids after D60 failed to obtain spheroids with smooth edges, no matter how long it took to repair (Figure S2B). Thus, the optimal cutting time window was defined as D35–D60. To investigate the effects of mechanical cut on cell damage and viability, we performed related assays 2 days after the mechanical cut. After the operation, 30 segmented organoids were cultured in one low‐adhesive 10‐cm dish, and the medium was supplemented to 15 mL. We changed the medium every day, and we found that the number of dead cells decreased after 48 h of culture (Figure S2C). At the same time, immunostaining results showed that the mechanical cut only affected the morphology of neurons (MAP2‐positive) on the cutting site, while the central area has morphological integrity (Figure S2D). After 14 days of reorganisation, immunofluorescence staining showed that the secondary generation of midbrain organoids retained the expression of SYP and MAP2, similar to their predecessors (Figure 2G,H). Meanwhile, cleaved caspase 3 (CC3)‐positive apoptotic cells were rapidly reduced in the segmented version (Figure 2I–K). Thus, mechanical splitting yielded small midbrain organoids with consistent cellular composition and less apoptosis.

### 3.3 Transcriptome Characterisation of Human Midbrain Organoids

**FIGURE 3:**
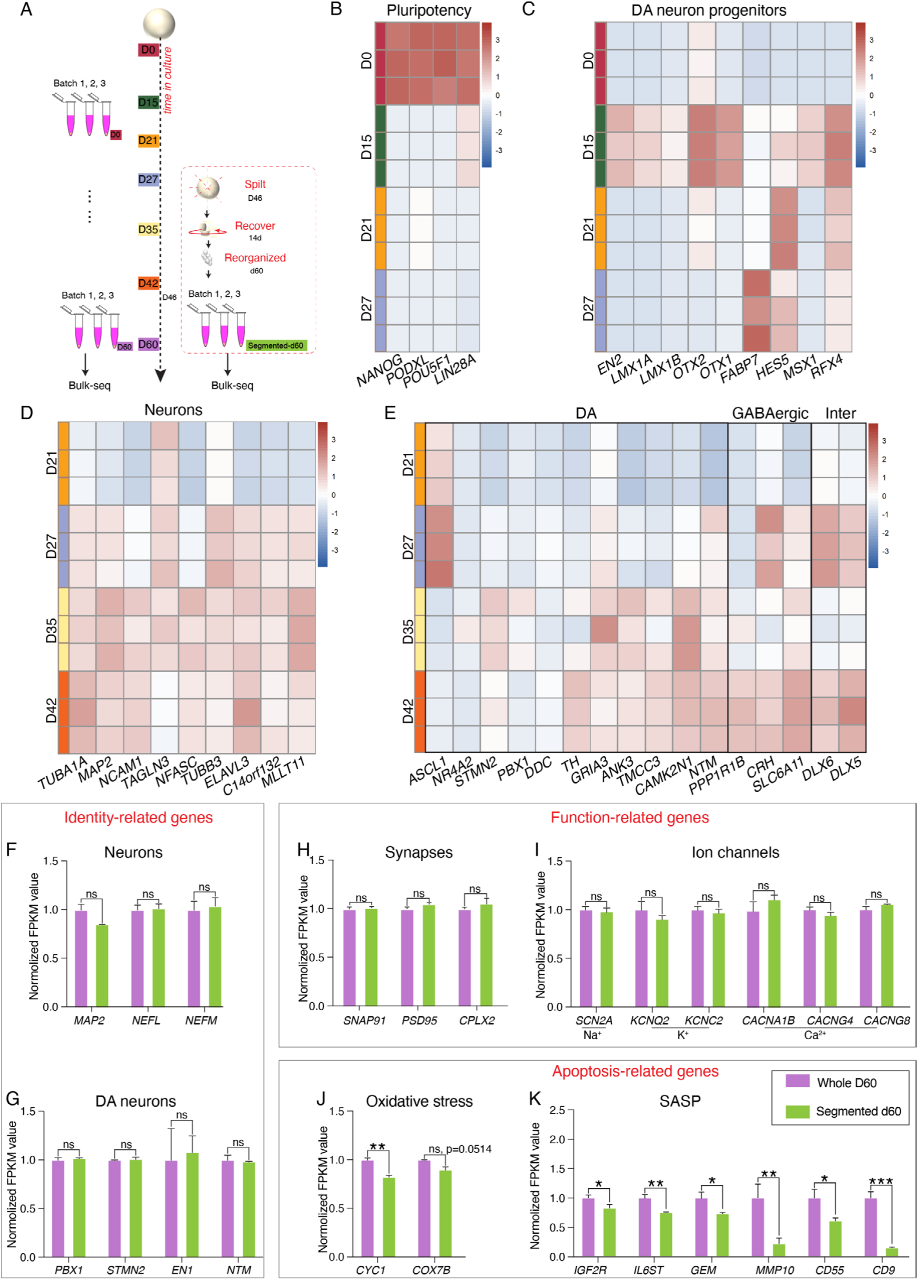
Transcriptome profiling of segmented and whole midbrain organoids. (A) Schematic of RNA sample collection at different timepoints. (B) Heat map showing expression of representative marker genes of pluripotency. (C) Heat map showing the relative mRNA expression of DA neuron progenitors during early midbrain organoids development. (D) Heat map depicting the relative mRNA level of neurons in each developmental timepoint. (E) Estimated cell abundance in matured midbrain organoids through deconvolution of bulk RNA sequencing transcriptomes. Inter, interneuron. (F–K) Comparison of FPKM values based on representative markers of neurons, DA neurons, ion channels, synapses, oxidative stress, and senescence‐associated secretory phenotype (SASP) between segmented and whole midbrain organoids. *n* = 3 organoids for each sample, two independent experiments. Data were shown as mean ± SEM. Two‐tailed unpaired *t*‐test. ns, no significance. *p < 0.05, **p < 0.01, ***p < 0.001.

To further characterise cell types and the impact of splitting, we performed bulk RNA sequencing (RNA‐seq) on organoids at critical time points and compared D60 midbrain organoids with secondary generation organoids (split at D46 and recovered for 14 days) (Figure 3A). From D15 to D27, pluripotent markers disappeared while DA neuron progenitor markers became prominent (Figure 3B,C). Neuronal markers such as *TUBB3* gradually increased from D21 (Figure 3D). Similar to in vivo DA neuron development, DA neuron‐specific genes such as *NR4A2* and *PBX1* [25, 26] were enriched from day 27 (Figure 3E).

Transcriptomic analysis revealed no differences in the expression of neuron, synapse, and ion channel‐related genes after splitting (Figure 3F–I). Splitting significantly reduced oxidative stress and senescence‐associated secretory phenotype (SASP) [27, 28] while maintaining neural function, consistent with the immunostaining results (Figure 3J,K). These findings confirmed that midbrain organoids expressed DA neuron‐specific marker profiles and that splitting did not compromise functional gene expression while reducing apoptosis‐related gene expression.

### 3.4 Formation of Neural Network in Human Midbrain Organoids

**FIGURE 4:**
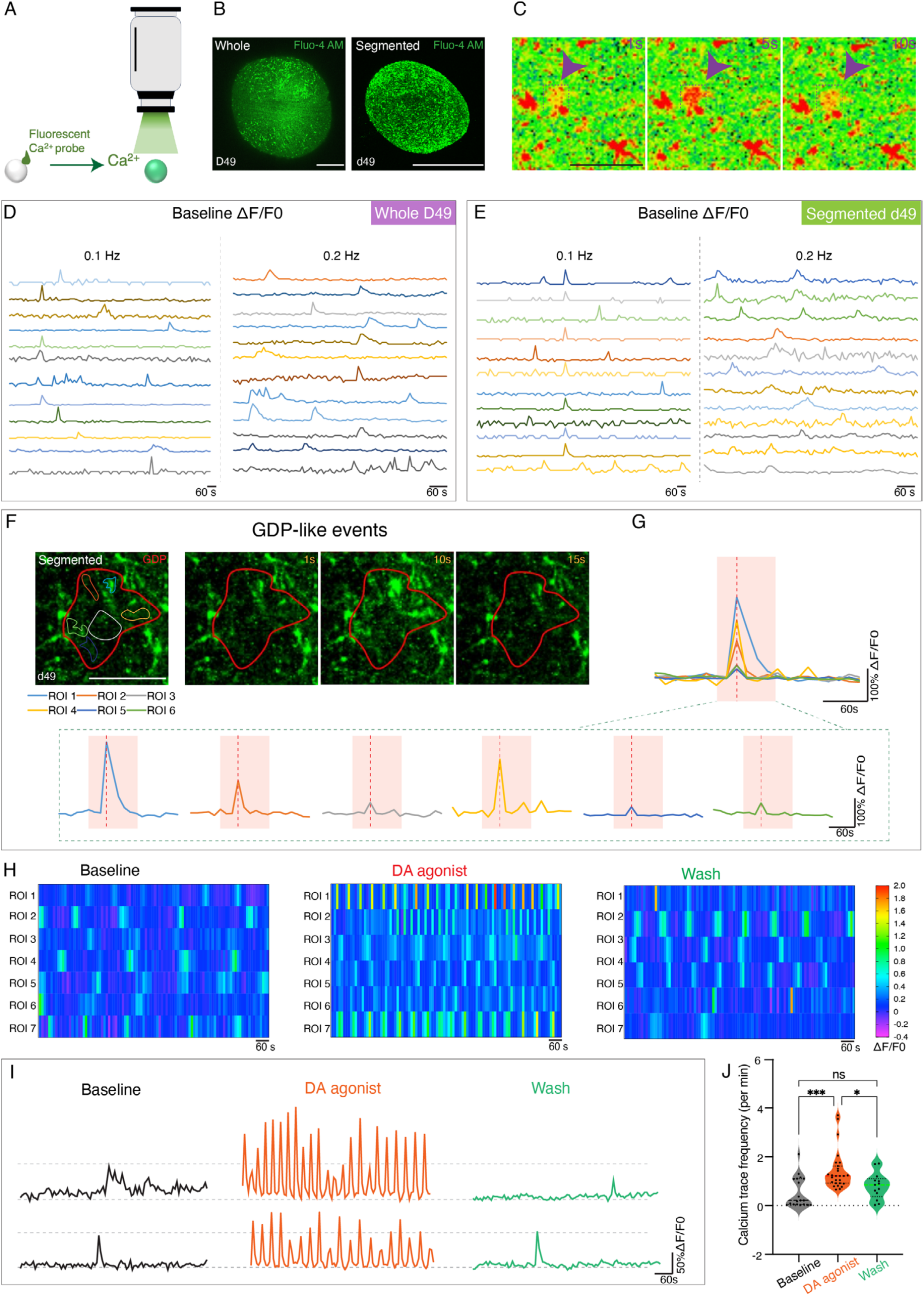
Segmented spheroids form similar neural network with that in ancestral midbrain organoids. (A) Summary of experimental procedures of calcium imaging. (B) Representative images of Fluo‐4AM‐labelled midbrain organoids and segmented organoids on day 49. Scale bar, 200 μm. (C) Representative images of regions of interest (ROIs) in two continuous time points. Scale bar, 50 μm. (D and E) Calcium traces under different sampling rates of midbrain organoids or segmented organoids on day 49. (F) Heatmap of ROIs and its giant depolarizing potential (GDP)‐like event of segmented midbrain organoids on d49. Scale bar, 50 μm. (G) Calcium traces of individual ROI contributed to one GDP‐like events. (H) Heatmaps of ΔF/F0 at baseline, DA agonist and wash states on d49. (I) Calcium traces of DA agonist‐reactive ROIs. (J) Quantification of calcium trace frequency at baseline, DA agonist and wash states. *n* = 60 ROIs from 3 organoids. Data were shown as mean ± SEM. One‐way ANOVA followed by Tukey's multiple comparison test. ***p = 0.0002, *p = 0.0308. ns, no significance.

We next investigated whether spontaneous and active neural networks were present in both organoids (Figure 4A). Calcium imaging revealed intracellular calcium transients, indicating functionally active neurons (Figure 4B,C). On day 49, calcium traces were uniformly recorded at different sampling frequencies, with 0.1 Hz yielding the most stable results for follow‐up experiments (Figure 4D,E). Time‐lapse imaging revealed giant depolarizing potential (GDP)‐like events and synchronous calcium responses across isolated regions, demonstrating well‐organised neural network (Figures 4F,G and S3A,B).

By introducing a DA agonist, we detected DA neuron‐specific calcium activity. Phenotypic changes in midbrain organoids included an increased frequency and ΔF/F0 value, possibly consistent with different DA neuron subtypes. Calcium activity returned to baseline following a medium rinse, confirming normal neural function (Figures 4H–J and S3C). Regardless of the splitting operation, both versions of midbrain organoids exhibited similar spontaneous and regular calcium traces.

### 3.5 Neurite Outgrowth and Electrophysiology In Vitro

**FIGURE 5:**
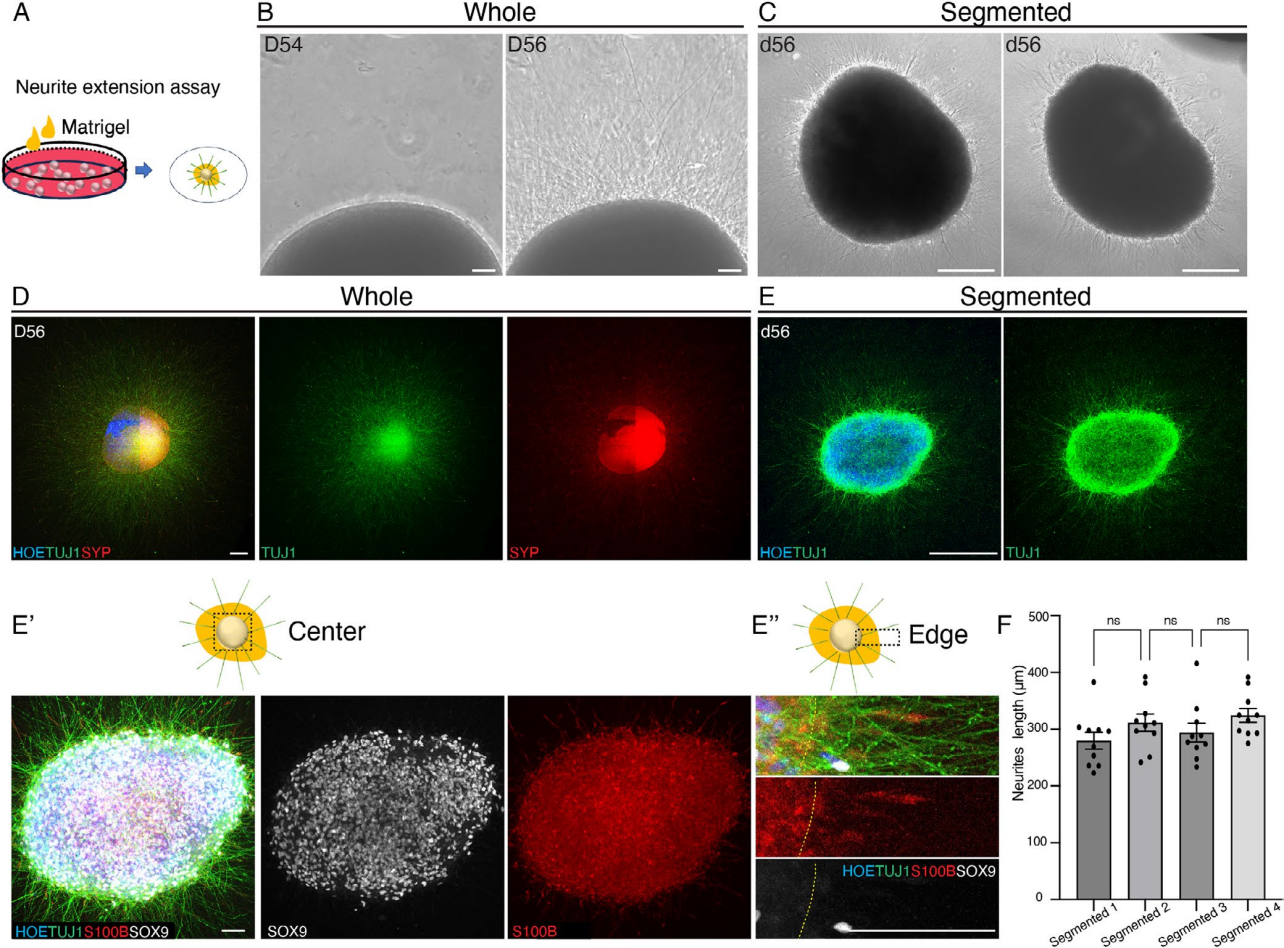
Segmented human midbrain organoids present similar neurites extension phenotype. (A) Summary of neurites extension experimental procedures. (B) Morphological changes of midbrain organoids. Scale bar, 200 μm. (C) Similar radial emission mode of neurites in segmented midbrain organoids with different shapes. Scale bar, 200 μm. (D) Immunostaining showing the neurites outgrowth of whole organoids on day 56. Scale bar, 200 μm. (E) Immunostaining showing the neurites outgrowth of segmented organoids on day 56. (E') Immunostaining of the center of segmented organoids showing the complete neurites network on the surface on d56. (E") Immunostaining of the edge of segmented organoids illustrating the positional relationship between glial cells (SOX9‐positive or S100B‐positive) and neurons (TUJ1‐positive) during the process of neurites outgrowth on d56. Scale bars, 200 μm (E), 50 μm (E', E"). (F) Quantification of the length of radiating neurons in different segmented organoids. Data were shown as mean ± SEM. One‐way ANOVA followed by Tukey's multiple comparison test. ns, no significance.

To simulate the neurite projection, Matrigel was introduced to the culture system of midbrain organoids (Figure 5A). Within 2 days, radiating neurites extended from both organoids, and the circularity of segments was negatively correlated with this phenomenon (Figure 5B,C,F). Neurites were SYP‐ and TUJ1‐positive, indicating neuronal maturity (Figure 5D,E). Glial cells marked by SOX9 and S100B were present at the organoid edges, possibly providing physical support (Figure 5E").

**FIGURE 6:**
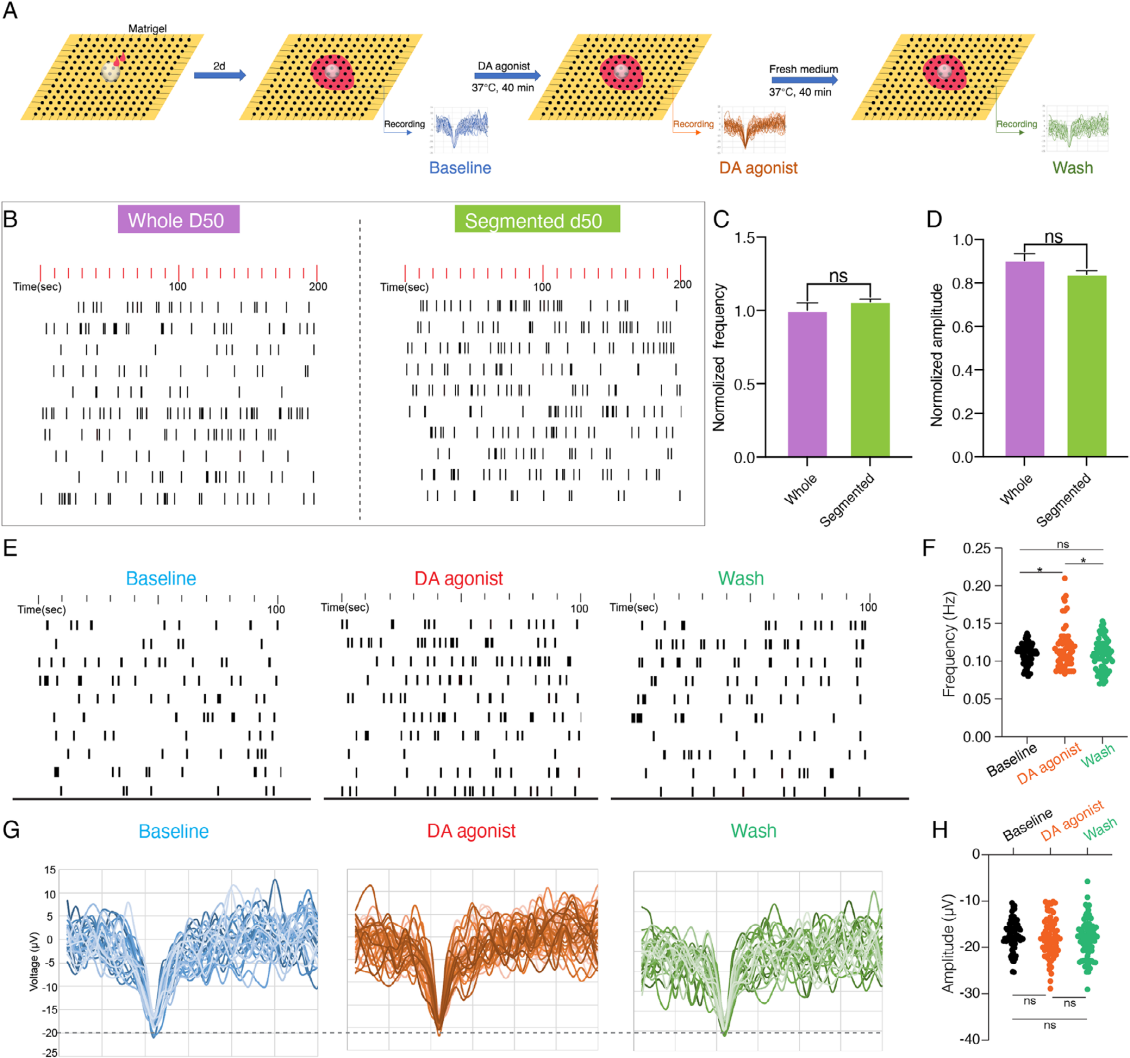
Segmented midbrain organoids preserve similar dopamine‐responsive electrophysiological activity. (A) Experimental set‐up to analyse neural electrical activities of midbrain organoids. (B) Frequency raster plots of 10 individual electrodes at baseline of whole and segmented organoids on day 50. (C and D) Quantification of spike frequency and amplitude in whole and segmented organoids. Data were shown as mean ± SEM. Two‐tailed unpaired *t*‐test. ns, no significance. (E) Representative frequency of segmented midbrain organoids under baseline and DA agonist stimulation. (F) Quantification of spike frequency under baseline and DA agonist states. *p < 0.05. ns, no significance. (G) Spike amplitude under baseline and stimulated states. (H) Quantification of spike amplitude under baseline and stimulated states. ns, no significance.

Electrophysiological activity was also assessed using MEA, which detected stable spontaneous field potential in D50 organoids (Figure 6A,B). Statistical analysis of frequency and amplitude remained largely stable further corroborated functional similarity between whole and segmented organoids (Figure 6C,D). DA agonists enhanced spike frequency without altering amplitude, and the increased activity returned to the baseline after media replacement (Figure 6E–H). These findings were consistent with calcium imaging results, highlighting the functional maturity of the organoids.

Overall, our data demonstrated the formation of a functional network in midbrain organoids, providing a robust platform for modelling DA neuron function and related disorders. These organoids offer significant potential for studying functional impairments and therapeutic interventions in neurological diseases such as PD.

## 4 Discussion

In this report, we present a fully scalable protocol for the generation, maturation, and functional characterisation of midbrain organoids. Our approach focused on optimising long‐term maintenance and evaluation assays for midbrain organoids, combining developmental patterning with mechanical cuts at appropriate stages. This protocol enables the generation of suitable midbrain organoids with functional DA neurons. Additionally, we developed a comprehensive set of functional assays, including calcium transient assay, neurite extension assay, and MEA assay, to evaluate neural network outcomes. These assays enable the sensitive detection of the effects of various insults on midbrain organoids.

Our protocol focused on midbrain DA neurons. Unlike protocols before, the composition detection and function assay of organoids are designed with DA neurons as the core, which can better reflect the development process and function evaluation of DA neurons. We generate midbrain organoids using hESCs for their broader cell fate potential to make sure the derived organoids are more consistent with brain development, while organoids using neuronal progenitors have relatively simple structures which might lack important developmental phenotypes [18]. This hPSCs‐based approach minimises variations in cell composition and volume control, and simplifies the experiment process, which is challenging to address in gel embedding‐based protocols [17, 26, 29]. The resulted midbrain organoids exhibit uniformity in size, cellular composition, gene expression, synchronous calcium activity, and electrical activity. These characteristics make them suitable for high‐throughput screening and unbiased, quantitative assessment in DA neuron‐related drug development. Furthermore, the midbrain organoids recapitulate key processes of DA neuron development, making them ideal for studying PD, particularly the pathogenesis of familial PD, and facilitating early intervention strategies targeting disease progression [18, 25, 30, 31].

Larger organoids increase the quantity of mature neurons but often lead to significant cell necrosis, particularly in the core regions [32]. While various methods have been developed to improve oxygen and nutrient supply, such as vascularising organoids in vitro or transplanting them into animal models, these approaches often lack practical applicability [21, 22, 33]. In our study, we adopted a straightforward method by dividing large organoids into smaller pieces, allowing them to resume growth. Within 2 weeks post‐operation, the divided pieces reorganised into spheric aggregates, with preserved cytoarchitectural characteristics and excluded apoptotic cells. Although this approach requires manual labour, the increased yield and improved organoid quality make it a practical and cost‐efficient solution.

Human brain functions rely on both complex organisational structures and the establishment of neural networks. Dysfunctional neural circuits are implicated in various brain diseases [9]. To evaluate the functional aspects of neural circuits within organoids, we introduced three assay methods. First, calcium imaging was employed to explore network‐level functions [34]. Distinct frequencies of oscillatory activity, synchronous GDP‐like events, and DA agonist responsiveness were observed, reflecting the development of the DA neural network. Second, organoids were seeded into Matrigel to promote neurite projection and neuronal migration, processes consistently observed in midbrain organoids between 35 and 60 days [35]. Third, the MEA system was used to record extracellular field potentials generated by the organoids. Field potential signals were captured at baseline, and DA responses were evidenced by enhanced frequency upon dopamine stimulation, consistent with previous studies [18, 36]. Together, these assays demonstrated that DA neurons in midbrain organoids receive environmental inputs, form functional neural networks, and reflect synaptic competence and connectivity. Reproducible results across different experimental batches underscore the robustness of these assays, positioning midbrain organoids as a reliable model for pharmacological and toxicological studies [37]. Meanwhile, by combining human induced pluripotent stem cells (hiPSCs) technology, hiPSC‐derived organoids could be used further in personalised medicine and targeting the underlying pathological process in young‐onset PD [38, 39].

In addition to PD, functional abnormalities of DA neurons affect various neurological diseases. Dysfunction of DA neurons in the nigrostriatal pathway will lead to abnormal motor behaviours and cognitive function, which may be associated with neurological disorders, such as Huntington's disease (HD) and schizophrenia [40, 41, 42, 43]. Impaired oxytocin signalling in DA neurons could alter behavioural responses to social novelty and cause autism spectrum disorders [44]. Neurotransmission in DA neurons is also involved in a wide range of attention deficit hyperactivity disorder (ADHD), depression, and cocaine addiction [45]. Thus, this midbrain organoid and functional assay method can be extended to other neurological abnormalities.

Despite limitations such as the absence of microglia and vasculature integration, the midbrain organoids generated in this study contain functional DA neurons, the electric manifestation of which is similar to their in vivo counterparts during midbrain development [46]. These organoids can survive long‐term, allowing for repeated and extended assessments. With further advancements to incorporate additional cell types and components, or through genetically engineering for specific purposes, these midbrain organoids and associated assay systems provide a practical yet powerful platform to advance basic research on brain development and disorders, and to facilitate the therapeutic development of diseases.

## Author Contributions

Xinyue Wang was involved in project design, data collection and analysis, and manuscript writing. Gaoying Sun supervised the project design, data processing and co‐wrote the paper. Mingming Tang analysed MEA data. Da Li maintained the normal operation of experimental equipment. Qi Jianhuan and Chuanyue Wang supplied constructive suggestions during data collection. Yukai Wang made key suggestions during the manuscript writing. Baoyang Hu conceived and supervised the project, provided instructive suggestions, and revised the manuscript.

## Conflicts of Interest

The authors declare no conflicts of interest.

## Supporting information


**Data S1** Supporting Information.

## Data Availability

The data that support the findings of this study are available from the corresponding author upon reasonable request.
