# Supplementary material for: Human Midbrain Organoids Enriched With Dopaminergic Neurons for Long‐Term Functional Evaluation
Source: Cell Prolif. 2025 Feb 20;58(7):e70005. doi: 10.1111/cpr.70005 (PMC12240635; doi:10.1111/cpr.70005)
Supplement: Supplementary file 1 — Data S1 Supporting Information. [file CPR-58-e70005-s001.docx]

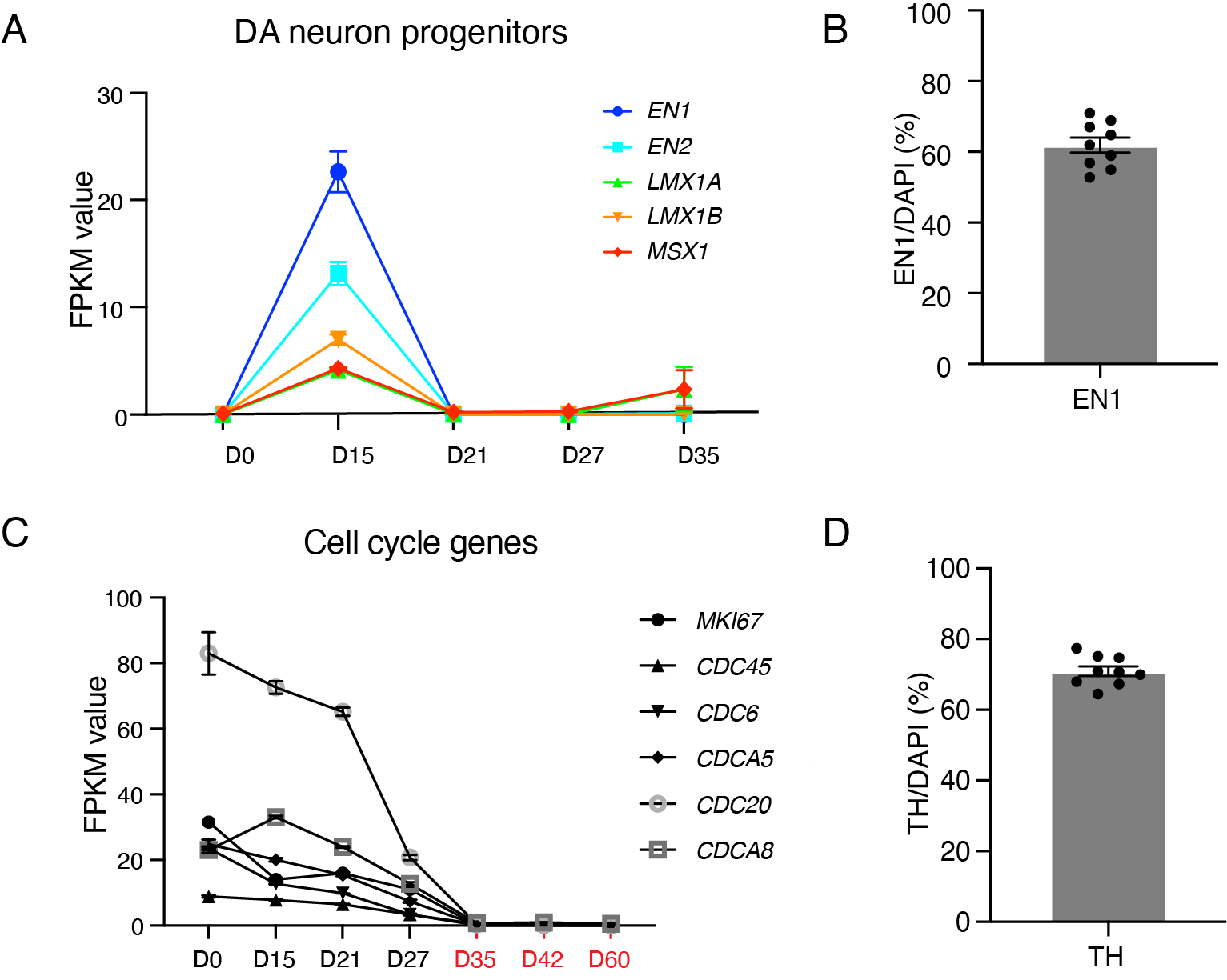


**Figure S1 Characterization of DA identity in human midbrain organoids during development.**

(A) Expression of DA neuron progenitor markers during D0 to D35. n=3~7 for each timepoint, 2 independent experiments. Data were shown as mean±SEM.

(B) Quantification of the expression of EN1-positive cells in D15. n=3, 2 independent experiments. Data were shown as mean±SEM

(C) FPKM values of cell cycle marker genes. *n*=3~7 for each timepoints, 2 independent experiments. Data were shown as mean±SEM.

(D) Quantification of the expression of TH-positive cells in D35. n=3, 2 independent experiments. Data were shown as mean±SEM.


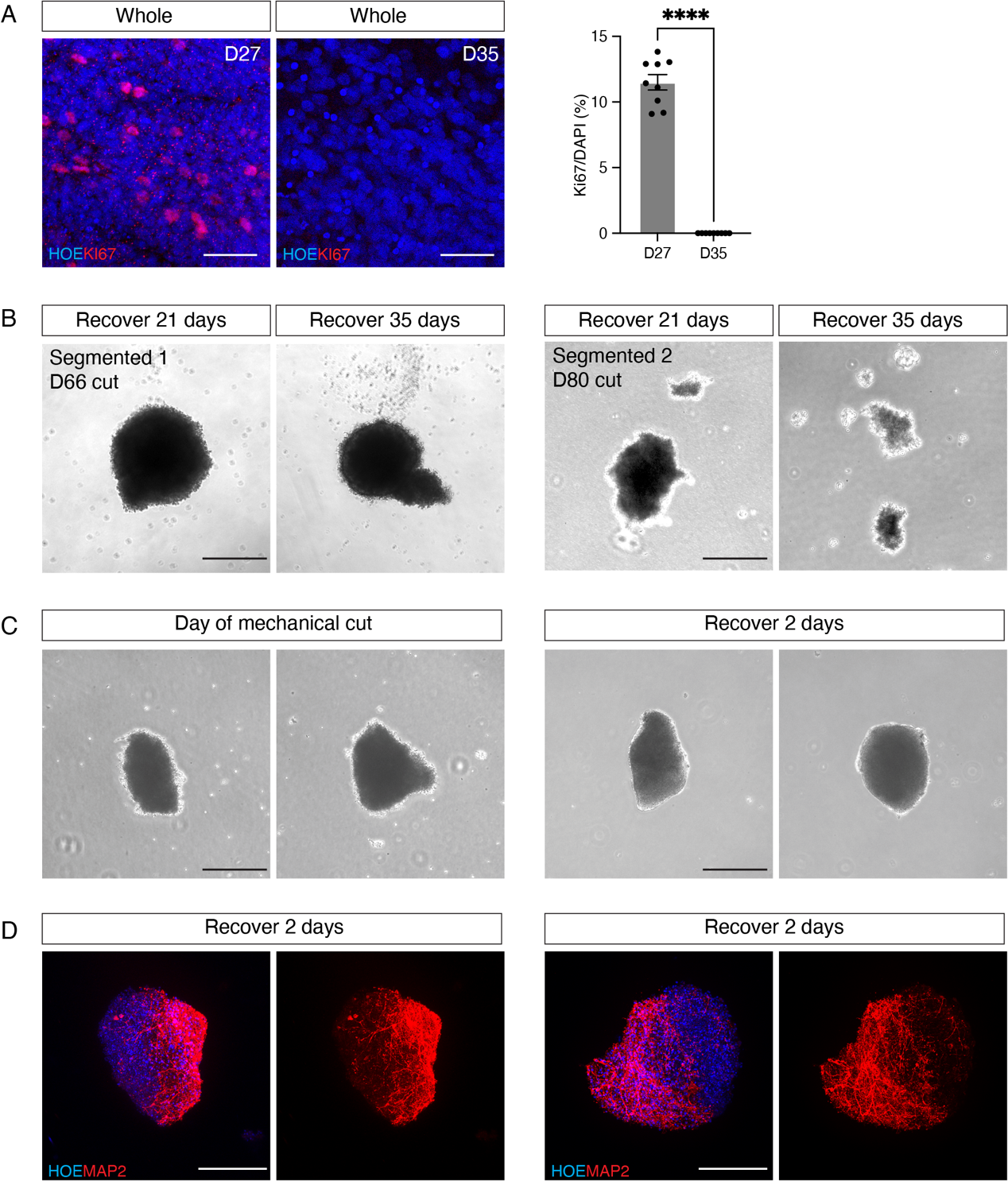


**Figure S2 The rationale for choosing optimal timepoints to mechanical cut.**

(A) Changes of proliferating cells marker (KI67) on D27 and D35. Scale bar, 50 µm. Data were shown as mean±SEM. Two-tailed unpaired t-test. *****P*<0.0001.

(B) Representative brightfield images of the repair process after the mechanical cut of whole organoids on D66 or D80. Scale bar, 200 µm.

(C) Representative brightfield images of the repair process in the short term after cutting. Scale bar, 200 µm.

(D) Representative immunostaining images of mature neurons (MAP2-positive) in the short term after cutting. Scale bar, 200 µm.


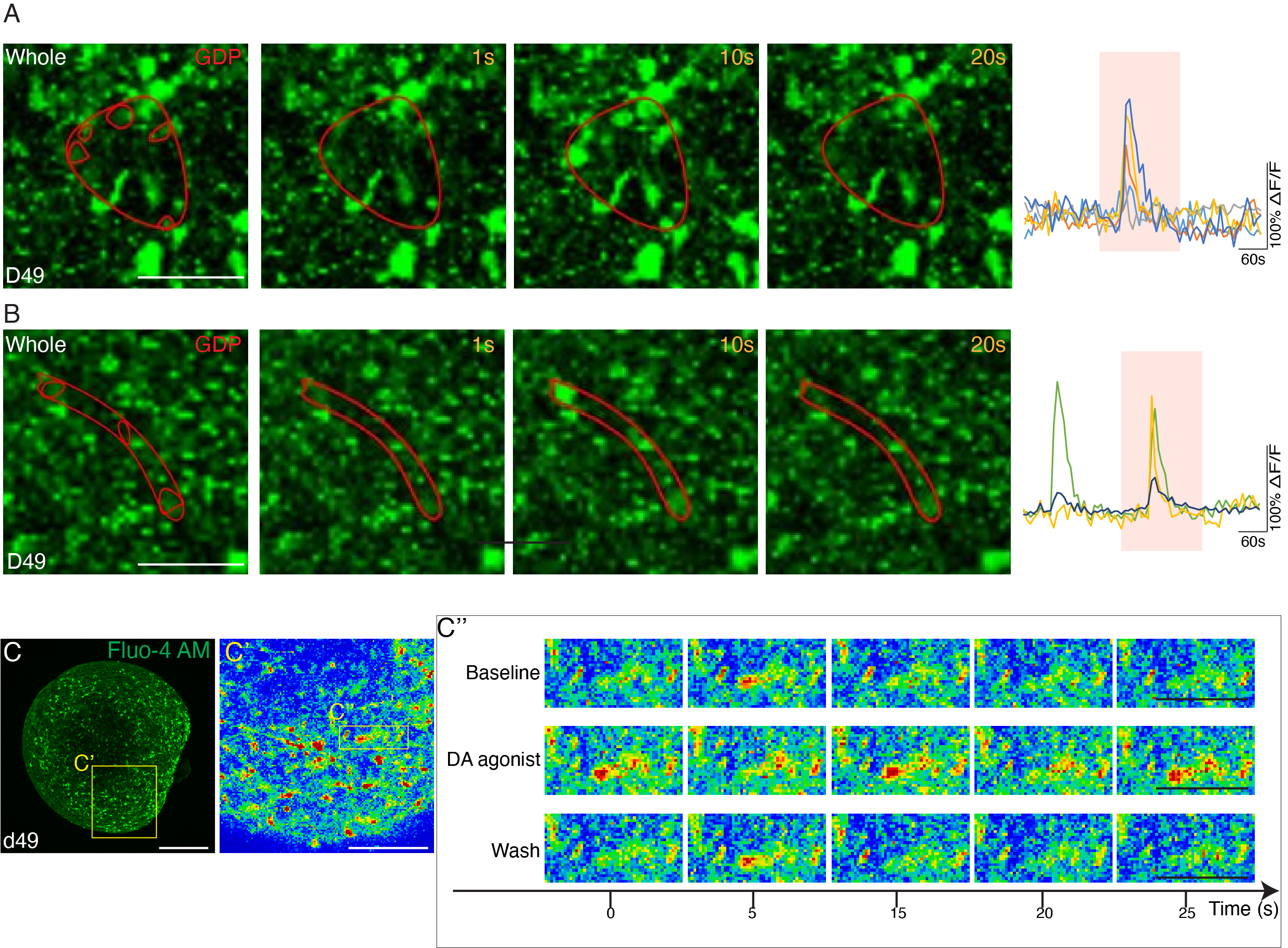


**Figure S3 Neural network in human midbrain organoids.**

(A-B) Images and calcium traces of different GDP-like events in continuous time points of whole organoids. A GDP-like event contained at least 3 individual ROIs. Scale bars, 50 µm (A, B).

(C) Image of Fluo-4 AM-labeled segmented organoids. C’, Heatmap of ROIs. C’’, Dynamic heatmaps of a calcium-loaded region in response to DA stimulation along time. Scale bars, 200 µm (C), 50 µm (C’, C’’).

**Supplementary Table 1. Antibodies**

| **Antibody** | **Host** | **Supplier** | **Catalog No.** | **Dilution** |
| --- | --- | --- | --- | --- |
| CC3 | Rabbit | CST | 9579S | 1:200 |
| ECAD | Mouse | Abcam | ab1416 | 1:500 |
| EN1 | Mouse | DSHB | 4G11 | 1:100 |
| LMX1A | Rabbit | Abcam | ab139726 | 1:200 |
| MAP2 | Chicken | Novus Biologicals | NB300-213 | 1:1500 |
| MAP2 | Mouse | Abcam | ab11267 | 1:500 |
| NCAD | Rabbit | Abcam | ab109737 | 1:200 |
| NESTIN | Mouse | Millipore | MAB5326 | 1:250 |
| OTX2 | Goat | R&D | AF1979 | 1:500 |
| S100B | Rabbit | Abcam | ab52642 | 1:500 |
| SOX1 | Goat | R&D Systems | AF3369 | 1:200 |
| SOX9 | Goat | R&D Systems | BAF3075 | 1:200 |
| SYP | Rabbit | Abcam | ab32127 | 1:500 |
| TH | Rabbit | Abcam | ab112 | 1:500 |
| TH | Rabbit | Abcam | ab152 | 1:500 |
| TH | Mouse | ImmunoStar | 22941 | 1:200 |
| TUJ1 | Mouse | Neuromics | MO15013 | 1:500 |
| TUJ1 | Mouse | Biolegend | 801201 | 1:1000 |
| TUJ1 | Rabbit | Biolegend | 802001 | 1:500 |
| VIM | Mouse | Abcam | ab8069 | 1:500 |
